# State Education

**Published:** 1881-10

**Authors:** Theophrastus Schopenhaver


### STATE EDUCATION.

BY THEOPHRASTUS SCHOPENHAVER.

I.

At other times and in divers places I have felt impelled to utter words of criticism upon our public school system ; but in accepting the invitation of my friend, the Editor, to expound briefly in the catholic columns of his BISTOURY, the theories which prompted, the experience which may have warranted such criticism, I had an other object in view. It is not my purpose in the series of articles hereby introduced to apply tests—either destructive or constructive in character—to the existing order of things, here or elsewhere. Nor do I contemplate theorizing merely. On the contrary, I propose to take for granted every possible consideration of a practical nature that can legitimately be urged by any of my readers. Thus, I shall assume that there is as yet no science of teaching, properly so called ; and that an overwhelming majority of parents have neither the time nor the mental and moral equipment—even if they felt the duty or inclination—themselves to instruct their children until these attain the "school going age;" and I do not propose to lay myself open to the charge of postulating either ideal scholars, parents, teachers or superintendence. No, we are poor, weak, foolish mortals, all of us—teacher and taught, it matters not which—let us however, instructed and persuaded by such eminently practical and successful educators as Pestalozzi, Frobel, Spencer and Baine, try to agree as to the *attainable* only, in the education of the masses, keeping our eyes fixed upon the desirable all the while.

Nor can it, accordingly, be my intention to scrutinize the data of history with a view to determining in how far the functions of family and church may have been arrogated by the State; or how in the course of time such allegedly illegitimate compulsion may have evolved into one of the chief, if not the principal functions of the body politic. Suffice it to say that in this XIX century, that in these United States of America—ignorance *is* vice, though it can no longer be said to possess exclusively the faculty of breeding the same. We must beware, lest harm befall the Republic, since its healthful growth, nay, its very stability, is dependent upon our eternal vigilance.

Now since the State does school our children ; and since for better or for worse this duty *must* under our system of government be a delegated one, from the irresponsible many to the accountable few, it concerns us to demand—in so far as they may not already have obtained vogue —the introduction of such a general system, and such particular methods as we may agree, lead to the most practical equipment of our boys and girls *in such branches of knowledge as we shall discover to be factors common to the various duties, and avocations of life. Such knowledge*, ALL OF IT, AND SUCH ALONE.

As good a definition, perhaps, as can be given of education is the following, in the words of Professor Mayhew: "Disciplining, culture, and furnishing of the mind of man, *qua* man, for his situation in life"—though I should change the order of steps to the following : 1, furnishing, 2, disciplining, 3, culture ; and it were very easy to adduce sufficient reasons for the same, had I not promised to eschew all theorrizing. That is :

In the first place, offer the child's mind facts, *facts*—and FACTS again ; not in a jumble, though, but in harmonious succession and complimentary order, so that gradually and without conscious effort the laws of their corelation and interdependence may dawn upon its understanding.

Next drill its memory by adequate, but none the less *practical* exercises. Thus the alphabet and multiplication table can be made the most useful of memnotic exercises in earliest childhood if properly taught.

And finally, after the perceptive and retentive faculties have thus all attained to some shape and dimension, their proper nourishment and growth must be the teacher's constant care.

Now in so far as my observation may enable me to judge all thoughtful educators of the day, are agreed on these points ; but when it comes to a practical application of the same to the needs of society as it is, I find that their systems become as numerous as the sands of the sea. In the face of this, my readers will not expect me to furnish still another panacea. Let us rather look things squarely in the face ; a little wisdom will, I am sure, teach us to put up with—nay to utilize even—the cussedness of inanimate things, material as well as of human nature. And as not he deserves the name of statesman, who, recognizing the evils and short-comings of society, proceeds straightway to evolve some scheme or "constitution" out of his individual consciousness, and then despairs in pessimistic disgust over the perverseness of human nature since he finds himself unable either to wheedle or terrorize his fellows into obeying the same, however enthusiastically and unanimously they may have "adopted" it, but he alone, who, utilizing the *status quo*, brings to pass great reforms and prodigious advances, despite the hatreds and sympathies, deep-rooted, unreasoning and unreasonable, of the masses and nations—by skillfully pitting wisdom against folly, virtue against vice, and *biding his time*; so is he but a fool who would regenerate the family and society *ab ovo* in order that his pet scholastic theory may become feasible. The true educator should be able to utilize schools as they are, and through them, in the course of time, approximate to the millennium so devoutly to be wished for. Again, therefore, let us take for granted.

First, that at least, ninety-nine hundredths of our children might as well have none of their senses at birth—so far as the education of their "perceptive faculties" are concerned;

Second, that in the public schools, to all intents and purposes, their memories alone are drilled; and

Third, that even "retentive faculties," being furnished *but little of practical consequence* to retain, can be but imperfectly cultured in the higher grades and schools.

Can my plea in view of this state of affairs, be other than,

1. *For the introduction of the Public Kindergarten*, as the indispensible foundation of the truly successful "grammar" school.

II. That *in the latter shall be taught language, numbers and the "science of common things"*—not the grammar, arithmetic and geography of the text books. And

III. That it is the duty of the State, that is to say, the duty of every community of the size and importance of Elmira, *to maintain a High School of such scope and equipment as will graduate competent teachers.*

To me these propositions seem incontrovertible in themselves; but it will be my endeavor in three successive articles, to lay before the readers of the BISTOURY, the facts and reasons which fortify them against all successful assault.

